# Left ventricular and atrial myocardial strain in heart failure with preserved ejection fraction: the evidence so far and prospects for phenotyping strategy

**DOI:** 10.1186/s12947-024-00323-1

**Published:** 2024-03-04

**Authors:** Mariane Higa Shinzato, Natasha Santos, Gustavo Nishida, Henrique Moriya, Jorge Assef, Fausto Feres, Renato A. Hortegal

**Affiliations:** 1 Dante Pazzanese Institute of Cardiology, São Paulo, SP Brazil Av. Dr. Dante Pazzanese, 500, 04012909; 2 Biomedical Engineering Laboratory, University of Sao Paulo, São Paulo, Brazil

**Keywords:** Heart failure with preserved ejection fraction, Speckle-tracking echocardiography, Left ventricular strain, Left atrial strain, Precision medicine

## Abstract

**Background:**

Heart failure with preserved ejection fraction (HFpEF) represents a significant proportion of heart failure cases. Accurate diagnosis is challenging due to the heterogeneous nature of the disease and limitations in traditional echocardiographic parameters.

**Main body:**

This review appraises the application of Global Longitudinal Strain (GLS) and Left Atrial Strain (LAS) as echocardiographic biomarkers in the diagnosis and phenotyping of HFpEF. Strain imaging, particularly Speckle Tracking Echocardiography, offers a superior assessment of myocardial deformation, providing a more detailed insight into left heart function than traditional metrics. Normal ranges for GLS and LAS are considered, acknowledging the impact of demographic and technical factors on these values. Clinical studies have demonstrated the prognostic value of GLS and LAS in HFpEF, especially in predicting cardiovascular outcomes and distinguishing HFpEF from other causes of dyspnea. Nevertheless, the variability of strain measurements and the potential for false-negative results underline the need for careful clinical interpretation. The HFA-PEFF scoring system's integration of these biomarkers, although systematic, reveals gaps in addressing the full spectrum of HFpEF pathology. The combined use of GLS and LAS has been suggested to define HFpEF phenogroups, which could lead to more personalized treatment plans.

**Conclusion:**

GLS and LAS have emerged as pivotal tools in the non-invasive diagnosis and stratification of HFpEF, offering a promise for tailored therapeutic strategies. Despite their potential, a structured approach to incorporating these biomarkers into standard diagnostic workflows is essential. Future clinical guidelines should include clear directives for the combined utilization of GLS and LAS, accentuating their role in the multidimensional assessment of HFpEF.

## Introduction

Heart failure with preserved ejection fraction (HFpEF) accounts for over one-half of all Heart Failure(HF) cases [1]. It is the most common form of HF, with its incidence and prevalence are growing as risk factors, including obesity, diabetes, and hypertension increase [2].

HFpEF is defined as the inability of the heart to pump blood properly at normal filling pressures. From a hemodynamic point of view, subjects with pulmonary arterial wedge pressure (PAWP) > 15 mmHg in rest conditions or > 25 mmHg during exercise have HFpEF [1]. However, the clinical definition of HFpEF varies substantially among studies, resulting in inconsistencies in non-invasive diagnosis [3–5].

The echocardiogram is a main non-invasive diagnostic tool that can provide several parameters to assess patients with suspected HFpEF. Even though the echocardiographic evaluation of diastolic function can provide parameters such as myocardial relaxation velocities and estimate the filling pressure non-invasively, those echocardiographic features present limited predictive capacities to diagnose HFpEF [2, 5].

The evaluation of left ventricular myocardial strain assessed by the Global Longitudinal Strain of the left ventricle (GLS) and Left Atrial Strain (LAS) have been proposed as robust and sensitive markers of left heart function with promising results to determine diagnosis and prognosis in HFpEF [6, 7]. Notwithstanding, there is a lack of data addressing the additive value of those echocardiographic features in the context of the recent recommendations to standardize the diagnostic criteria.

This review discusses whether the impressive evidence base for GLS and LAS justifies their use as biomarkers of HFpEF.

## Technical considerations of strain imaging

### Definition of strain

**Fig. 1:**
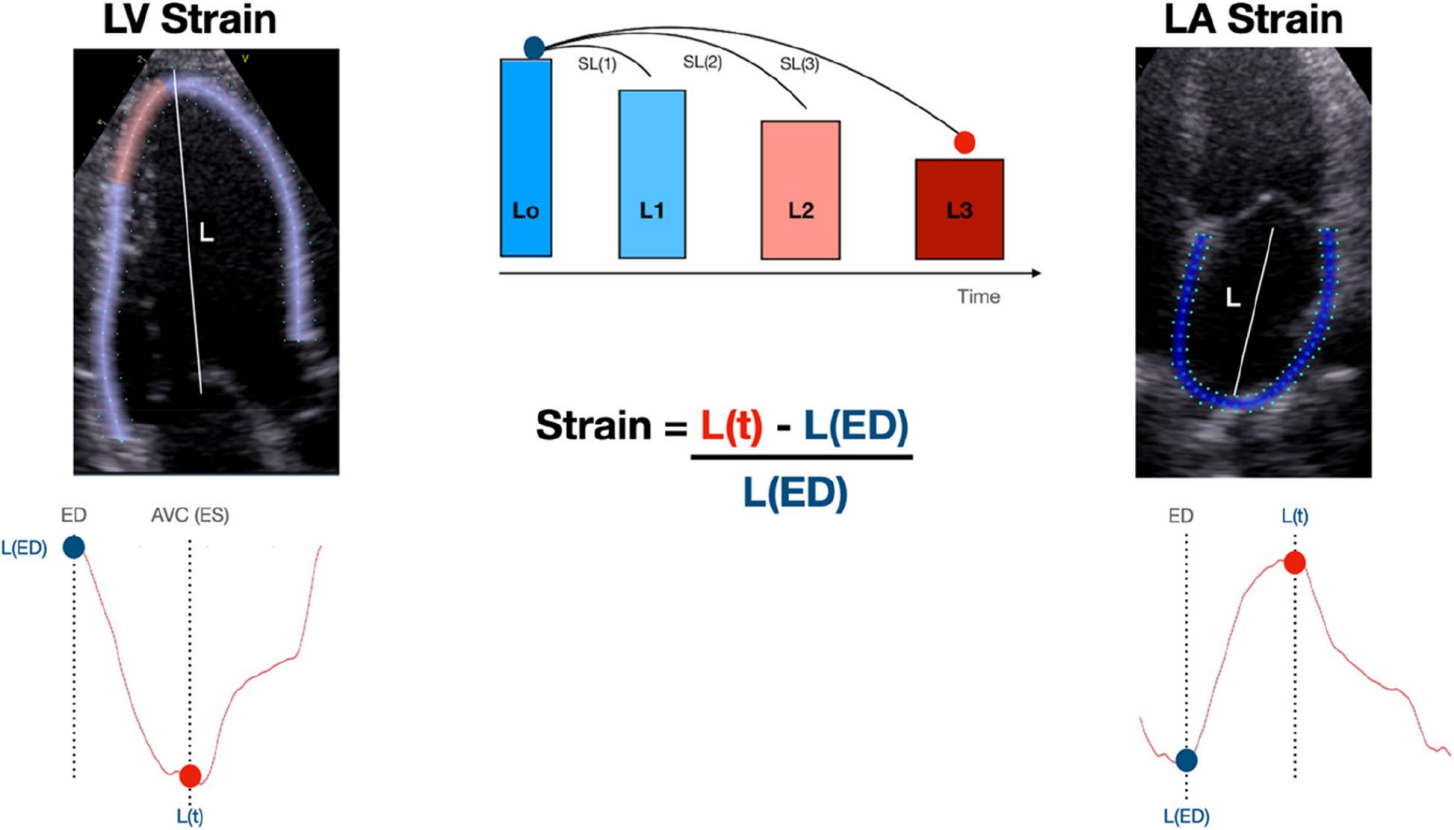
The concept of Left Ventricular Strain (LV Strain) depicted on the left side and Left Atrial Strain (LA Strain) depicted on the right side represent the relative changes in chamber length over a specified time interval -L(t) (depicted by red full circle), in comparison to the chamber length in end-diastole, denoted as L(ED) (depicted by blue full circle). The L(ED) is often defined as the time at which the mitral valve closes or the automatic detection of peak QRS occurs. For LV Strain, the time ‘t’ corresponds to end-systole (ES), which is precisely defined as aortic valve closure (AVC). On the other hand, for LA strain, the reservoir phase corresponds to the zenith of the strain curve

Strain corresponds to the amount of deformation of an object in relation to its original form. In Cardiology, this concept is represented as the percentage (%) of shortening/lengthening of the heart in relation to its initial measurement [7, 8]. Mathematically, the Lagrangian strain is computed by the algorithm as strain(t) = 100 [L(t) – L(ED)/L(ED)], where L(t) is the longitudinal length at time t, and L(ED) is the end-diastolic length [9] (Fig. 1). Significant differences exist between software regarding the L(ED) length used: an entire line of the ROI x average of a certain number of ROI points x average of the values in each segment of the same frame [9].

The Speckle Tracking technique has become the most recommended technique to estimate cardiac strain due to its better reproducibility and lower sensitivity to the ultrasound insonation angle, despite having lower temporal resolution when compared to Tissue Doppler Imaging [9].

The use of the concept of strain to assess myocardial function has been validated experimentally in vivo using sonomicrometry and cardiac magnetic resonance [10].

### Left ventricular strain: definition and normal range

The GLS reflects the relative longitudinal contraction (%) of the myocardium that occurs from the period of isovolumetric contraction until the end of the ejection period [9, 11].

The GLS normality value is approximately 20%. There is evidence of variations in normality values according to sex and age. Absolute values of GLS ranging from 16–18% are frequently stated as borderline, while an absolute below 16% indicates systolic dysfunction.

*Farsalinos *et al. [12] demonstrated that the greatest absolute difference between manufacturers for GLS values was 3.7 percentage units of strain, with a significant and strong correlation between the measurements of the different manufacturers and also with the average measurement of all the manufacturers. Furthermore, caution is required regarding software updates, which can also impact GLS calculations.

Despite these findings, GLS is an echocardiographic feature that is less susceptible to technical factors than ejection fraction assessments by conventional 2D echocardiography, and it can be applied in daily practice [7, 13].

### Left atrial strain: definition and normal range

Analogous to the left ventricle, LAS is a metric that quantifies the dynamic changes in the length of the atrial myocardium, indicating both shortening and lengthening, across the cardiac cycle. It serves as a valuable indicator of LA deformation. LAS proves to be an important tool with prognostic value for a broad spectrum of cardiovascular diseases [14]. Imaging in the far ultrasound field and a thinner left atrial (LA) myocardium may pose additional challenges to LAS analysis. For the same reason, assessment of radial strain or a subdivision of the LA wall into segments is not recommended [15]. Several studies have demonstrated the use of echocardiographic LAS to evaluate LA mechanical function [16–18].

The LAS comprises three distinct phases: reservoir, conduit, and contractile. The reservoir phase signifies the stretching of the LA wall resulting from the filling of the left atrium through pulmonary veins while the mitral valve is closed. The conduit phase takes place in the early diastole when the mitral valve opens, and the LA discharges into the left ventricle, corresponding to the E wave (early transmitral flow). The contractile phase corresponds to the A wave (late transmitral flow) and denotes the contraction of the left atrium. Each component of the LA is represented by a LAS element: LAS reservoir (LASr), LAS conduit (LAS cd), and LAS contractile (LASct). In the LAS curve, each phase can be represented by a measure obtained through the difference in values at two points [15]. Based on actual evidence, LASr has the most important clinical utility due to its prognostic and morbimortality prediction.

Reference values for LA function parameters using strain have been investigated in various publications. A meta-analysis [19] comprising 40 studies and involving 2542 healthy patients defined the following normal values: 39.4% (95% CI: 38.0–40.8%) for LASr, 23% (95% CI: 20.7–25.2%) for LAScd, and 17.4% (95% CI: 16.0–19.0%) for LASct. Heterogeneity was attributed to sample size, heart rate, and body surface area. Age, gender, racial groups, reference zero for strain, and differences between software types were not identified as contributors to the variation.

Due to the wide range of reference values for LAS compared to LV strain, the lower limit of normality demonstrated has been considerably lower than the average values described.

Results from the EACVI NORRE study [20], which included 371 healthy patients, indicated the following absolute normal values and lower expected values (indicated in parentheses) for LA strain: 42.5% (26.1%) for reservoir function, 25.7% (12.0%) for conduit function, and 16.3% (7.7%) for contractile function.

*Nyberg *et al. [21], based on 1329 healthy individuals, described the values of 17%, 3%, and 7% as the lower limits of normality for LASr, LAScd, and LASct, respectively.

Given the variability in reference ranges for LAS, caution should be exercised when incorporating it into clinical decision-making in various contexts.

## Evidence of left ventricular and atrial myocardial strain in HFpEF

### The left ventricular global longitudinal strain in HFpEF

The role of GLS in HFpEF has been assessed in animal models and clinical studies. Despite all the challenges related to producing an animal model that recapitulates all major features of HFpEF syndrome, recent data has provided new insightful findings. Using a model of chronic hypertension in rats, *Shah *et al. [22] shows that GLS could be a biomarker in transitioning from adaptive cardiac hypertrophy to dysfunction toward cardiac failure. The abnormalities in strain imaging seem to happen when there are changes in transverse (T)-tubule organization. This leads to altered intracellular Ca2 + cycling, abnormal excitation–contraction coupling, and the development of abnormal myocardial mechanics. These changes seem to manifest prior to the development of significant cardiac fibrosis and precede the development of overt cardiac dysfunction and HF, indicating that impairments in GLS may have a role in pre-clinical HF (pre-HFpEF, previously called stage B). These findings are in agreement with clinical studies suggesting that impairments in GLS may occur in early-stage HFpEF. For example, *Kosmala *et al. [23] demonstrated that in asymptomatic patients with type 2 diabetes mellitus, impaired GLS is independent and incremental to left ventricular hypertrophy in predicting incident HF.

Clinical studies also have evaluated GLS in patients with overt HFpEF syndrome. A large cohort study [24] demonstrated that an abnormal GLS was strongly associated with a more than twofold increase in the composite endpoint of cardiovascular (CV) death, hospitalization due to heart failure, or aborted cardiac arrest. This association remained significant even after adjusting for risk factors and other echocardiographic features.

*Sakaguchi *et al*. *[23] demonstrated the enduring prognostic significance of GLS changes in HFpEF patients. Assessing GLS during acute HF hospitalization and in a stable phase, they observed that patients with major cardiovascular events (MACE) had a significantly lower GLS change rate than those without MACE (10,6% vs. 26%; *p* < 0.001), highlighting impaired GLS as a robust prognostic indicator for a more adverse disease course.

In the context of recent efforts to standardize diagnostic criteria such as the H_2_FPEF and HFA-PEFF scores, there is a notable gap in data addressing the additional value of Global Longitudinal Strain (GLS). The H_2_FPEF score [25] does not incorporate GLS, making it challenging to assess its contributory significance based on existing evidence.

The HFA-PEFF score, introduced by European guidelines in 2019 [26] and posteriorly validated in two large cohorts [24], presents a systematic framework for diagnosing patients suspected of HFpEF. While this score provides a valuable evaluative metric, physicians should be aware of the potential for false negatives, which could account for up to 25% [27], necessitating a careful application of the score alongside comprehensive clinical assessments to ensure diagnostic accuracy.

**Fig. 2:**
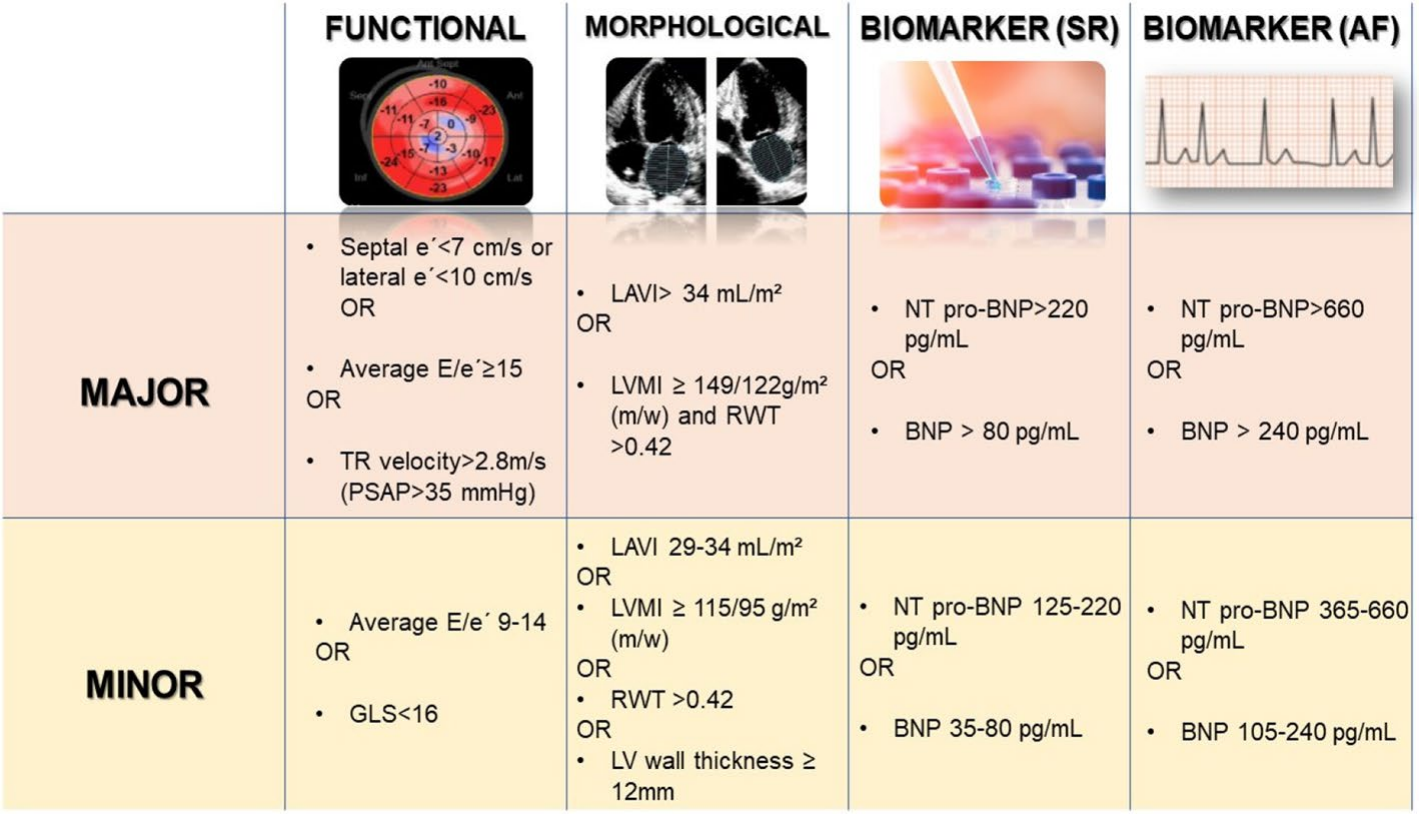
Echocardiographic and natriuretic peptide heart failure with preserved ejection fraction workup and scoring system. TR: Tricuspid Regurgitation. LAVI: Left Atrial Volume Index. LVMI: left ventricular mass index. RWT: Relative Wall Thickness. M: Men. W: Women. SR: Sinus Rhythm.AF: atrial fibrillation. Adapted from: Pieske, B et al. How to diagnose heart failure with preserved ejection fraction: the HFA–PEFF diagnostic algorithm: a consensus recommendation from the Heart Failure Association of the European Society of Cardiology

The acronym “PEFF” represents the following sequential steps: Pre-test assessment (P), Echocardiography (E), Functional evaluation (F1), and Final etiology determination (F2). During Step E, the echocardiographic parameters are assessed alongside plasmatic biomarkers (Fig. 2), where, GLS is categorized as a minor parameter within the functional domain. Thus, not all patients with abnormal GLS will have HFpEF.

The abnormal GLS has been associated with conditions that are known to affect the myocardium, such as hypertension, diabetes mellitus, subclinical atherosclerosis, aortic stenosis and amyloidosis, These conditions can be considered as risk factors or even as HFpEF mimics [7, 9].

This abnormal GLS without others pathophysiological impairments may not be sufficient to lead to an increase in LV filling pressures. Indeed, there is evidence pointing that GLS have only a moderate correlation with the time constant of LV pressure decay, tau [28], and with resting pre-A pressure [29].

Since patients with HFpEF may exhibit increased filling pressures exclusively during exercise, studies addressing the behavior of GLS during hemodynamic stress such as exercise, preload challenge and afterload challenge can potentially have relevant information. Recent data reported the myocardial dysfunction mediated by increased afterload in patients with HFpEF with preserved resting GLS [30]. Those subjects displayed an accentuated decrease in the Longitudinal Strain during provoked pressure overload.

Furthermore, other aspects of LV mechanics beyond the GLS, such as the degree of temporal heterogeneity of contraction (mechanical dyssynchrony), are more associated to exercise capacity in HFpEF patients and may provide new insights on how the heart could be more prone to the steep increase filling pressures during exercise that characterized HFpEF [31].

These data support the notion that overt HFpEF can occur without impaired resting GLS, indicating that the isolated use of GLS may have sub-optimal sensitivity in the HFpEF population. Indeed several studies have substantial proportion of patients with fully developed HFpEF with preserved GLS [32] as shown in Table 1.

**Table 1:** Some studies analyzing the GLS thresholds in patients with HFpEF

Trial	Strain Analysis (N)	GLS normal	GLS abnormal	Cut off for abnormal GLS
TOPCAT [33]	447	48%	52%	-15.8%
RELAX [34]	187	35%	65%	-16%
Donal et al. [35]	237	33%	67%	-16%
Buggey et al. [36]	739	24%	76%	-16%
PARAMOUNT HF [37]	219	33%	67%	-15.8%
Huang et al. [38]	129	24.1%	75.9%	-15.8%
Morris et al. [39]	119	18.5%	81.5%	-16%

Adapted from: Argulian E, et al. [32]. *GLS *Left Ventricular Global Longitudinal Strain

### The left atrial strain in HFpEF

The LA strain has emerged as a valuable echocardiographic parameter in the assessment of HFpEF, offering insights into the pathophysiology and prognostication of this complex syndrome [39–44]. The role of LA strain in HFpEF is multifaceted, primarily focusing on its correlation with LV filling pressures, major adverse cardiovascular events (MACE), and the risk of atrial fibrillation (Table 2).

**Table 2:** Evidence for left atrial strain in patients with HFpEF

**Invasive Hemodynamic outcomes**	Published trials
LVEDP	Cameli et al., 2016 [45] Lin et al., 2020 [46] Ma et al., 2022 [47] Zhou et al., 2021 [48]
PAWP	Reddy et al., 2019 [49] Lundberg et al., 2019 [50] Telles et al., 2019 [51]
LV pre-A pressure	Singh et al., 2019 [52]
LV pre-A pressure/PAWP	Inoue et al., 2021 [53]
LVEDP/PAWP	Hummel et al., 2017 [54]
LVEDP/LV pre-A pressure	Nishida et al., 2023 [41]
**Clinical outcomes**	
All-cause/cardiovascular mortality or HF hospitalization	Park et al., 2020 [55] Bouwmeester et al., 2022 [56] Ersbøll et al., 2013 [57] Kim et al., 2020 [58] Inciardi et al., 2022 [59] Shin et al., 2021 [60] Oike et al., 2021 [61]
Atrial fibrillation	Jasic-Szpak et al., 2021 [62] Weber et al., 2021 [63]

Adapted from *Nagueh *et al. [64]. Legend: *LVEPD* Left Ventricular End Diastolic Pressure, *PAWP* Pulmonary Arterial Wedge Pressure, *LV* Left Ventricular, *pre-A* pre-atrial contraction, *HF* Heart Failure

#### Correlation with LV Filling Pressure (PAWP)

LA strain serves as a non-invasive marker for assessing LV filling pressures, often reflected by pulmonary artery wedge pressure (PAWP). Abnormalities in LA strain, especially in reservoir phase, have shown consistent correlation with elevated LV filling pressures, aiding in the diagnosis and monitoring of HFpEF patients. A decrease in LV filling pressures is associated with a reduction in LA volumes, although normalization is infrequent. Notably, a robust correlation exists between the reduction in LV filling pressure and the enhancement of LA function, as evidenced by improvements in LA strain [43]. This correlation is particularly crucial as elevated filling pressures are a hallmark of HFpEF and contribute to its clinical manifestations.

#### Association with major adverse cardiovascular events (MACE)

LA strain has demonstrated predictive value for MACE in HFpEF patients. Impaired LA strain is associated with adverse cardiovascular outcomes, including all-cause mortality, cardiovascular mortality, and HF hospitalizations. The compromised LA function, as reflected by reduced strain, highlights the intricate interplay between LA dysfunction and the progression of HFpEF. As a result, LA strain becomes a valuable prognostic tool, aiding clinicians in risk stratification and identifying patients at a higher risk of cardiovascular events.

#### Risk of Atrial Fibrillation (AF)

HFpEF patients frequently exhibit a high prevalence of atrial fibrillation, a condition associated with increased morbidity and mortality. LA strain has shown promise in predicting the development of AF in HFpEF cohorts. Impaired LA function, as assessed by reduced strain values, is associated with atrial remodeling and electrical disturbances, contributing to the initiation and perpetuation of AF. Therefore, LA strain not only aids in assessing the current state of HFpEF but also serves as a potential marker for identifying individuals at an increased risk of developing AF.

In this context, LA strain plays a pivotal role in HFpEF management by offering valuable insights into LV filling pressures, predicting major adverse cardiovascular events, and identifying patients at risk of atrial fibrillation. As an integral component of advanced echocardiographic assessment, the LAS has been shown to be the most robust imaging marker distinguishing HFpEF from noncardiac causes of dyspnea, and abnormalities in LAS are more associated with adverse outcomes than those of LV [39, 40].

Both H_2_FPEF and HFA-PEFF scoring systems do not incorporate the potential insights provided by LAS. The LASr improves the feasibility and diagnostic accuracy of the 2016 ASE/EACVI diastolic algorithm in patients with HFpEF and, so, can furnish additional information that aids in the diagnosis of HFpEF. Furthermore, the LAS reservoir has proven aid to identify those patients with normal resting filling pressure who subsequently developed elevated LVFP during exercise [40].

## Proposal for the combined use of GLS and LAS to Phenotype HFpEF patients

Recently it was proposed the potential Phenotyping of HFpEF syndrome according to the abnormalities in LV strain [32, 65]. Abnormalities in left ventricular longitudinal strain have been identified as a valuable marker for distinguishing a specific phenogroup within the diverse spectrum of HFpEF syndrome. This phenogroup consists of individuals with reduced GLS (HFpEF-rLS), indicating the presence of contractile dysfunction, myocardial fibrosis, maladaptive myocardial hypertrophy, and other myocardial diseases [7, 13, 66]. These underlying mechanisms contribute to the alteration of GLS in HFpEF.

Conversely, a notable proportion of HFpEF patients (ranging from 18 to 48%) exhibit preserved LV longitudinal systolic function (HFpEF-pLS) [32, 67]. This subgroup may represent a distinct phenogroup that has progressed toward other major pathophysiological abnormalities such as atrial dysfunction, chronotropic incompetence, pulmonary vascular disease, unbalanced blood volume distribution, and low peripheral oxygen extraction, which can combine in different permutations, ultimately contributing to the development of heart failure syndrome.

In this context, given the growing body of research providing high-quality, evidence-based support for LAS as a robust biomarker in Heart Failure with Preserved Ejection Fraction (HFpEF) and Left Atrial (LA) myopathy, the integration of LAS into the assessment of patients with HFpEF-pLS appears promising and holds an excellent pathophysiological rationale. Furthermore, this additional value seems to manifest independently of the presence of persistent or paroxysmal atrial fibrillation, thereby amplifying both diagnostic and prognostic insights.

In patients with HFpEF-pLS and reduced LAS, there is a disproportionate impairment within the left atrium chamber due to atrial cardiomyopathy [65], establishing the left atrium as a pivotal mechanical epicentrum in the pathophysiology mechanism of the disease. The evidence supporting this rationale is relatively new in the field of Cardiology, as most classical knowledge about diastology assumes LA disease as a direct consequence of advanced LV diastolic dysfunction with chronic increase in LV end-diastolic pressure [68].

Recent data suggests that despite having better LV diastolic function, these patients exhibit a worse hemodynamic profile characterized by higher pulmonary artery pressure and pulmonary vascular resistance, coupled with a lower stroke volume [65]. Additionally, they are more prone to displaying abnormalities in LV strain during exercise, further supporting the evidence of an association with microvascular dysfunction.

With a more pronounced degree of impairments in LA function, these patients may demonstrate reduced LV filling, leading to a decreased LV end-diastolic volume. Consequently, they may exhibit heart failure with a supranormal LVEF profile [69, 70].

**Fig. 3:**
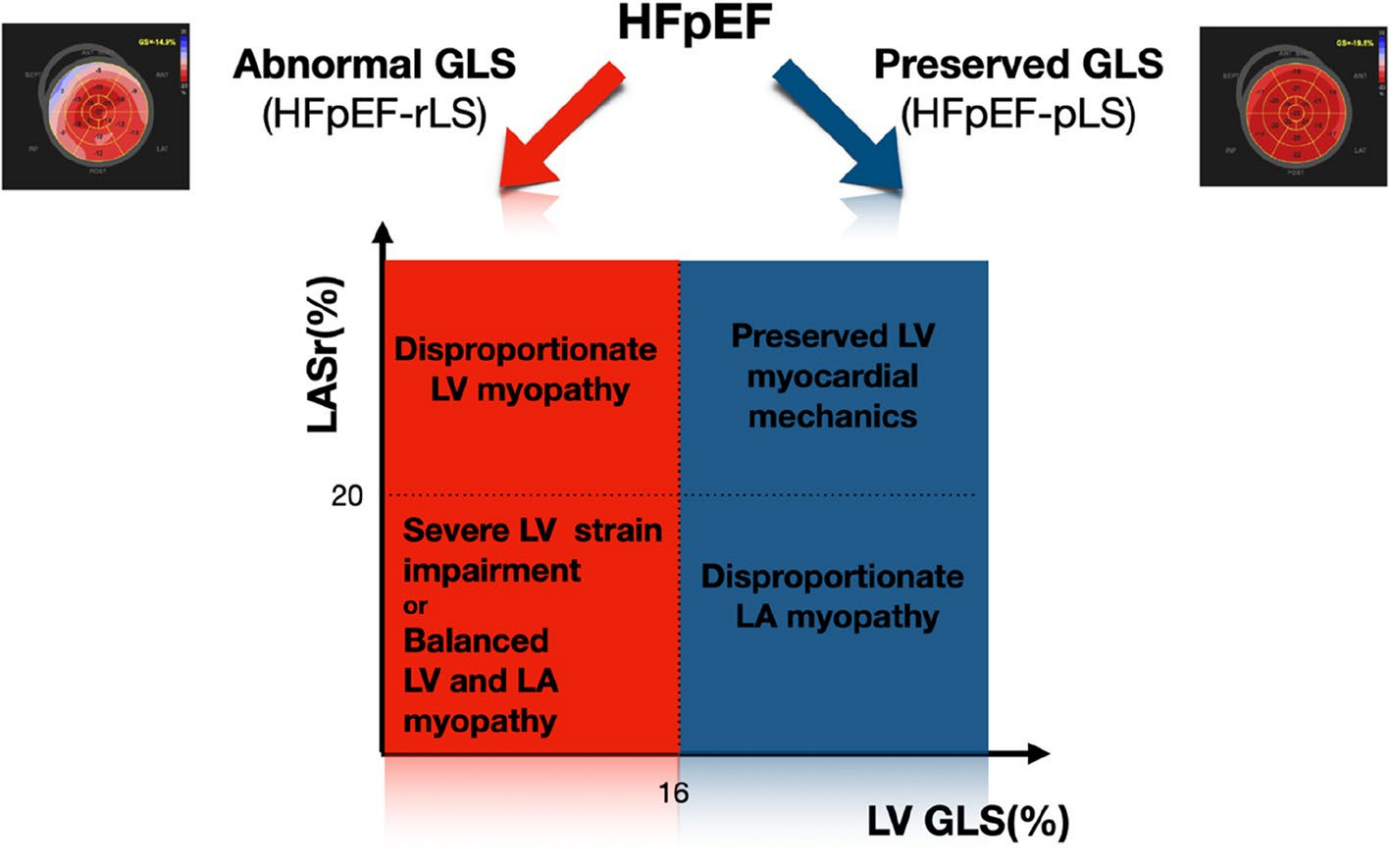
Proposed methodology for phenotyping Heart Failure with Preserved Ejection Fraction (HFpEF) through parameters derived from left heart myocardial mechanics. There are two mains distinct HFpEF phenotypes based on left ventricular strain: HFpEF with reduced longitudinal systolic function (HFpEF-rLS) and HFpEF with preserved longitudinal systolic function (HFpEF-pLS). Additionally, the integration of the left atrium strain reservoir (LASr) is suggested to introduce a comprehensive dimension addressing the impairments in both the left ventricle and left atrium. This proposed approach offers a nuanced perspective for refining HFpEF phenotyping and may contribute to targeted therapeutic insight

The concurrent application of both GLS and LAS for phenotyping subjects with HFpEF is depicted in Fig. 3. This proposal strategy has the potential to provide a more comprehensive insight into the specific cardiac impairments of HFpEF patients. Moreover, it holds promise for the development of innovative randomized controlled trial designs such as enrichment trials [3].

This type of trial design can test interventions to specific sub-phenogroups. This type of study represents one of the main tools to deal with HFpEF heterogeneity since the traditional randomized controlled trial design may have significant limitations in this scenario [3].

## Conclusions

The current evidence substantiates the utility of GLS and LAS for diagnostic of HFpEF. Their combined use offers a promising approach to phenotyping patients with HFpEF, potentially guiding more personalized therapeutic interventions. While compelling, the evidence calls for a carefully structured integration of GLS and LAS into standard diagnostic protocols to fully leverage their potential in clinical practice.

## Data Availability

None.

## Abbreviations

ACC: American College of Cardiology
AF: Atrial Fibrillation
AHA: American Heart Association
CV: Cardiovascular
EF: Ejection Fraction
GLS: Global Longitudinal Strain
HF: Heart Failure
HFA: Heart Failure Association
HFpEF: Heart Failure with Preserved Ejection Fraction
HFpEF-pLS: Heart Failure with preserved Ejection Fraction and preserved Longitudinal Systolic function
HFpEF-rLS: Heart Failure with preserved Ejection Fraction and reduced Longitudinal Systolic function
LAS: Left atrial strain
LASr: Left atrial strain reservoir
LAScd: Left atrial strain conduit
LASct: Left atrial strain contractile
LAVI: Left Atrial Volume Index
L(ED): End-Diastolic Length.
LV: Left Ventricular
LVH: Left Ventricular Hypertrophy
LVMI: Left Ventricular Mass Index
MACE: Major Cardiovascular Events
NP: Natriuretic Peptides
PAWP: Pulmonary Wedge Pressure
RWT: Relative Wall Thickness
SP: Speckle Tracking
SR: Sinus Rhythm
TR: Tricuspid Regurgitation
